# 2013, a very good year

**DOI:** 10.11604/pamj.2014.17.72.3918

**Published:** 2014-01-30

**Authors:** Robert Davis

**Affiliations:** 1 American Red Cross, International Services, Nairobi, Kenya

**Keywords:** Child survival, MDG, EPI, polio maternal health, malaria

## Introduction

Reviewing evidence from health and other areas, the writer Zack Beauchamp concluded that “2013 was the best year in human history” [1]. His focus was broad and societal; the present discussion will focus on progress in health issues, with a special emphasis on the Millennium Development Goals. With 2015 only two years away, the two most watched Millennium Development Goals on health are MDGs 4 and 5, respectively targeting under-five mortality and maternal mortality. Both are down by 47 percent since 1990. This is what the UN had to say about under-five mortality in September 2013.

## Under-five mortality reduction

**From MDG 4: reduce by two thirds, between 1990 and 2015, the under-five mortality rate [2]**

- Despite population growth, the number of deaths in children under five worldwide declined from 12.4 million in 1990 to 6.6 million in 2012, which translates into about 17,000 fewer children dying each day.
- Since 2000, measles vaccines have averted over 10 million deaths.
- Despite determined global progress in reducing child deaths, an increasing proportion of child deaths are in sub-Saharan Africa where one in ten children die before the age of five and in Southern Asia where one in 16 die before age five.
- As the rate of under-five deaths overall declines, the proportion that occurs during the first month after birth is increasing.
- Children born into poverty are almost twice as likely to die before the age of five as those from wealthier families.
- Children of educated mothers—even mothers with only primary schooling—are more likely to survive than children of mothers with no education.

The years since the turn of the century have focused on specific measures aimed at quick returns in bringing down under-five mortality. Without neglecting targeted interventions, it may now be time to make more investments in such areas as female education, which produces benefits over the medium and long term in reducing both under-five and maternal mortality.

## Maternal mortality reduction

Progress on MDG5 [3] has been less speedy, with declines in maternal mortality less rapid than planned. Achieving durable declines in maternal mortality means the creation or build-up of district hospital obstetrical services in urban and, especially, rural areas. Ingenious solutions are still needed for the problem of transporting the obstetrical emergency cases from remote areas to the hospital. Especially in parts of Africa, the proportion of skilled deliveries has to go up. These items mean financing for running costs, which will, in general, have to come from governments. So the future of maternal mortality reduction is inextricably linked to higher levels of financing for health in the public sector.

**Target 5.A: Reduce by three quarters the maternal mortality ratio**

- Maternal mortality has nearly halved since 1990. An estimated 287,000 maternal deaths occurred in 2010 worldwide, a decline of 47 per cent from 1990. All regions have made progress but accelerated interventions are required in order meet the target.
- In Eastern Asia, Northern Africa and Southern Asia, maternal mortality has declined by around two-thirds.
- Nearly 50 million babies worldwide are delivered without skilled care.
- The maternal mortality ratio in developing regions is still 15 times higher than in the developed regions.
- The rural-urban gap in skilled care during childbirth has narrowed.

**Target 5.B: Achieve universal access to reproductive health**

- More women are receiving antenatal care. In developing regions, antenatal care increased from 63 per cent in 1990 to 81 per cent in 2011.
- Only half of women in developing regions receive the recommended amount of health care they need.
- Fewer teens are having children in most developing regions, but progress has slowed.
- The large increase in contraceptive use in the 1990s was not matched in the 2000s.
- The need for family planning is slowly being met for more women, but demand is increasing at a rapid pace.
- Official Development Assistance for reproductive health care and family planning remains low.

Birth spacing remains the Cinderella of global health, much discussed [4] but little funded, despite its proven impact on child and maternal mortality. The UN Population Fund has been its strongest advocate in recent years, but UNFPA is working almost alone in promoting birth spacing.

Among the other MDGs, reduction in gender disparities [5] is perhaps the most relevant to future progress in reducing maternal and under-five mortality. In a *Lancet*discussion of “What's happening in Bangladesh,” Amartya Sen looks at the reduction in gender disparities and other factors which account for this poor country's posting better MDG performance than countries at the same economic level [6]. Writing in the same journal, Adams and colleagues review the factors leading to Bangladesh's rapid declines in child mortality [7].

## HIV/AIDS

The year 2013 saw progress on both the preventive and therapeutic side in the fight against HIV/AIDS. The report “Towards an AIDS Free Generation” [8] quotes the following figures:

- 62 percent of pregnant women living with HIV in the 22 Global Plan priority countries received antiretrovirals to prevent mother-to-child transmission in 2012.
- Among children (0–14 years) in low- and middle-income countries, 850,000 new HIV infections were prevented between 2005 and 2012.
- AIDS-related deaths among adolescents (10–19 years) increased by 50 percent between 2005 and 2012.
- Overall, deaths fell by 30%.

The UNAIDS 2013 publication, AIDS by the Numbers [9], shows progress on all fronts:

- 33% decrease in new HIV infections since 2001
- 29%decrease in AIDS-related deaths (adults and children) since 2005
- 52% decrease in new HIV infections in children since 2001
- 40-foldd increase in access to antiretroviral therapy 2002–2012

Globally, the number of new HIV infections continues to fall. There were 2.3 million new HIV infections (1.9 million–2.7 million) in 2012. This is the lowest number of annual new infections since the mid-to-late 1990s, when approximately 3.5 million (3.3 million–4.1 million) people were acquiring HIV every year.

The cost of first line antiretroviral therapy in some low and middle-income countries has been reduced to around US$ 140 per person per year. In the mid 1990's the cost was around US$ 10 000 per person per year. In 2012 alone an additional 1.6 million people newly gained access to treatment.

On the institutional side, 2013 saw the publication of the 10 year evaluation, by the Institute of Medicine, of PEPFAR, a US government initiative to curb the spread of AIDS in Africa. With a favorable evaluation, and bipartisan support, PEPFAR is likely to continue, even in today's atmosphere of fiscal stringency.

## Malaria

The year end World Malaria Report brought good news in malaria control, as this WHO summary revealed: “Global efforts to control and eliminate malaria have saved an estimated 3.3 million lives since 2000, reducing malaria mortality rates by 45% globally and by 49% in Africa, according to the “World malaria report 2013” published by WHO.

An expansion of prevention and control measures has been mirrored by a consistent decline in malaria deaths and illness, despite an increase in the global population at risk of malaria between 2000 and 2012. Increased political commitment and expanded funding have helped to reduce incidence of malaria by 29% globally, and by 31% in Africa.

The large majority of the 3.3 million lives saved between 2000 and 2012 were in the 10 countries with the highest malaria burden, and among children aged less than 5 years – the group most affected by the disease. Over the same period, malaria mortality rates in children in Africa were reduced by an estimated 54%.

. . . In 2012, there were an estimated 207 million cases of malaria (uncertainty interval: 135 – 287 million), which caused approximately 627 000 malaria deaths (uncertainty interval 473 000 – 789 000). An estimated 3.4 billion people continue to be at risk of malaria, mostly in Africa and south-east Asia. Around 80% of malaria cases occur in Africa.

. . . In sub-Saharan Africa, the proportion of the population with access to an insecticide-treated bed net remained well under 50% in 2013. Only 70 million new bed nets were delivered to malaria-endemic countries in 2012, below the 150 million minimum needed every year to ensure everyone at risk is protected. However, in 2013, about 136 million nets were delivered, and the pipeline for 2014 looks even stronger (approximately 200 million), suggesting that there is real chance for a turnaround.

There was no such setback for malaria diagnostic testing, which has continued to expand in recent years. Between 2010 and 2012, the proportion of people with suspected malaria who received a diagnostic test in the public sector increased from 44% to 64% globally.” [10]

Among the challenges which remain for malaria control and eradication:

- Closing the funding gap, which has in recent years led to a relative stagnation in bednet coverage. The GFATM replenishment meeting just held in Washington was a step in this direction
- Dealing with the growing threat of insecticide resistance and drug resistance
- Incorporating the first licensed malaria vaccine (likely to become available in or after 2015) into national programs

## Expanded Programme On Immunization

As EPI approaches its 40th birthday – the resolution creating EPI passed in May 1974 – the number of vaccines available in most developing countries has doubled from the 1970s. The vaccines now available, but not 40 years ago, protect against hepatitis B, H. influenzae B, cervical cancer, rotavirus, rubella, and pneumococcal disease. A new vaccine against Type A meningitis is being rolled out in the meningitis belt of Africa, with initially gratifying results. A promising malaria vaccine awaits licensing in or after 2015. An oral cholera vaccine is now available which gives better protection than the legacy injectable product.

The vehicle for delivery of both new and old vaccines is routine immunization. Here, progress has been uneven at best. As the 2013 Assessment Report on the Global Vaccine Action Plan noted,

At the current pace, many countries—mainly in the African, Eastern Mediterranean and South-East Asia Regions—will not meet routine immunizations coverage targets: to achieve coverage with routine immunization of 90% or greater at the national level and 80% or greater in every district. More worrying is that immunization coverage has remained low, stagnant or even decreasing in several of these countries. Countries with low and stagnant coverage for routine immunization must urgently intensify efforts to improve programme performance, utilizing administrative data and surveys to direct their corrective actions. Civil society needs to be meaningfully engaged in policy dialogues so that reasons for low coverage are better understood and interventions are accepted and tailored to address identified problems. Countries, agencies and all development partners must engage with the vaccine industry to closely monitor the global supply of vaccines and ensure sufficient supply into the future. They should anticipate and take timely actions to mitigate the risks of vaccine supply shortfalls that contribute to low coverage [11].

The tools for raising routine immunization, such as the RED approach (reach every district) are well known. The human and financial resources to raise routine are lacking, and will be until national and external funding for routine becomes more widely available.

Within EPI, three diseases are currently targeted for global elimination or eradication: tetanus, measles, and polio. While 2013 saw some progress in tetanus elimination and measles control, polio has gone over a rough patch. Here are the 2012 and 2013 figures as of mid-December (Table 1) [12].

**Table 1:** Wild Poliovirus Cases, Globally, 2012 and 2013

	Year to date, 2013	Year to date, 2012	Total, 2012
Total	372	222	222
In endemic countries	148	216	217
In non-endemic countries	224	6	6

Source: www.polioeradication.org (14 January 2013)

Wild poliovirus persists in all three endemic countries, though at somewhat lower levels than in 2012. Epidemics in Somalia and neighboring countries constitute a large setback, as do more recent outbreaks in Syria. The Independent Monitoring Board for the global polio initiative wrote as follows in its most recent report.

*Unprecedented challenges loom over the polio eradication program. There is shocking violence to which no public health program should ever be subjected. Bans prevent the program from vaccinating two million children against polio in Pakistan and Somalia. The program has dealt with insecurity before (and continues to do so) but these are different phenomena. All who support the eradication of the second ever disease for humankind should have no greater priority than seeking to resolve them*.

*The program has far from perfect control in such circumstances. Whilst we are sympathetic to the challenge that this creates, it is more important than ever that the program's performance be as eradication-ready – as worthy of a global public health emergency – as it can be in the many aspects that are within its control*.

*There are too many instances in which this is not the case. The performance issues to be addressed are illustrated by (but not limited to) the fact that the Horn of Africa was not better protected against an outbreak and that too many other countries remain vulnerable. They are illustrated too by the response in the Horn of Africa, which could not be described as a robust response to a public health emergency of global health importance. It is also important to realise that too many suboptimal campaigns continue in each of Afghanistan, Nigeria and Pakistan, even in areas where insecurity is not a major feature*.

*As the program enters what is supposed to be the last low season in which polio circulates, we ask ourselves (as should all within the program): it this a program that is eradication-ready? Does what we are seeing really look like a programmatic emergency for global public health? Is the leadership and chain of command properly aligned to the challenges of today? This report identifies too many ways in which this is not the case*.

*The goal of stopping polio transmission by the end of 2014 now stands at serious risk. This situation must be turned round with the greatest possible urgency*.

## Perspectives for 2014 and beyond

As the MDG deadlines of 2015 approach, the world community has to look not only at what has been done, but what remains to be done. This means a careful review of the context within which the MDGs live and breathe. In particular, is health a priority? Is it enough of a priority to assure ceasefires and corridors of peace? As of this writing, global initiatives against polio and measles are at risk in Somalia, South Sudan and the Central African Republic because health is not a priority to the combatants. This must change.

So, too, must the distribution of resources within governments. When Paul Kagame came to power in Rwanda, one memo on his desk was an urgent request for ministerial luxury cars. That request was cancelled. Resources were reallocated to the social sector. Today, landlocked Rwanda, with few mineral resources, is doing better at vaccination and malaria control than any of the oil producing giants of Africa. Resources matter, but so do resource allocation decisions.

After 2015, the post-MDG goals need to reflect the extent to which governments put their money where their mouths are. Measures of transparency, good governance, and transfer of resources into the social sector are the underpinnings of post-2015 progress in health and all other sectors.
